# Origin of the mysterious Yin-Shang bronzes in China indicated by lead isotopes

**DOI:** 10.1038/srep23304

**Published:** 2016-03-18

**Authors:** Wei-dong Sun, Li-peng Zhang, Jia Guo, Cong-ying Li, Yu-hang Jiang, Robert E. Zartman, Zhao-feng Zhang

**Affiliations:** 1 CAS Key Laboratory of Mineralogy and Metallogeny, Guangzhou Institute of Geochemistry, Chinese Academy of Sciences, Guangzhou 510640, China; 2 CAS Center for Excellence in Tibetan Plateau Earth Sciences, Chinese Academy of Sciences, Beijing 100101 China; 3 State Key Laboratory of Isotope Geochemistry, Guangzhou Institute of Geochemistry, Chinese Academy of Sciences, Guangzhou 510640, China

## Abstract

Fine Yin-Shang bronzes containing lead with puzzlingly highly radiogenic isotopic compositions appeared suddenly in the alluvial plain of the Yellow River around 1400 BC. The Tongkuangyu copper deposit in central China is known to have lead isotopic compositions even more radiogenic and scattered than those of the Yin-Shang bronzes. Most of the Yin-Shang bronzes are tin-copper alloys with high lead contents. The low lead and tin concentrations, together with the less radiogenic lead isotopes of bronzes in an ancient smelting site nearby, however, exclude Tongkuangyu as the sole supplier of the Yin-Shang bronzes. Interestingly, tin ingots/prills and bronzes found in Africa also have highly radiogenic lead isotopes, but it remains mysterious as to how such African bronzes may have been transported to China. Nevertheless, these African bronzes are the only bronzes outside China so far reported that have lead isotopes similar to those of the Yin-Shang bronzes. All these radiogenic lead isotopes plot along ~2.0–2.5 Ga isochron lines, implying that deposits around Archean cratons are the most likely candidates for the sources. African cratons along the Nile and even micro-cratons in the Sahara desert may have similar lead signatures. These places were probably accessible by ancient civilizations, and thus are the most favorable suppliers of the bronzes.

An important archaeological artifact for understanding the early cultural and technological development of China is the splendid Yin-Shang bronze wares dating back to ca. 1400 BC. These objects, representing a significant advancement in metal working, appeared suddenly at that time in the alluvial plain of the Yellow River, in Henan Province, central China, and also from several places in southern China at roughly the same time or slightly earlier. However, the source of the copper, lead, and tin ores used to manufacture the bronze remains obscure, especially when attempting to explain the radiogenic isotopic compositions of their lead^1^,^2^ and the paucity of the required tin deposits within the territory of Yin-Shang in central China.

Ancient books and myths suggested that China has a long civilized history of ca. 5000 years or longer^3^ although to many archaeologists, this is yet to be firmly established. The Shang civilization is one of the pillars of the history of China. Yin-Shang is the last period of the Shang dynasty according to oracle bone inscriptions unearthed in Anyang and the famous history book, “Shih Chi”^3^. It is generally taken by western archaeologists as the earliest advanced civilization in China. Large quantities of fine bronzes and oracle bones, together with composite bows and chariots, have been found in Yin-Shang ruins, all of which are the earliest of their kinds so far discovered in China^4^,^5^.

## Bronze with Unique Lead Isotopes

Yin-Shang bronzes mark a peak in the late Bronze Age all over the world, with a variety of fine bronze wares, e.g., the 832.84 kilogram giant Houmuwu Ding bronze vessel of 133 centimeter height, 110 centimeter length, and 79 centimeter width. Many of the bronze wares have names and/or stories written on them in ancient Chinese characters, providing key evidences for the well-developed Yin-Shang bronze civilization and history of China.

**Figure 1:**
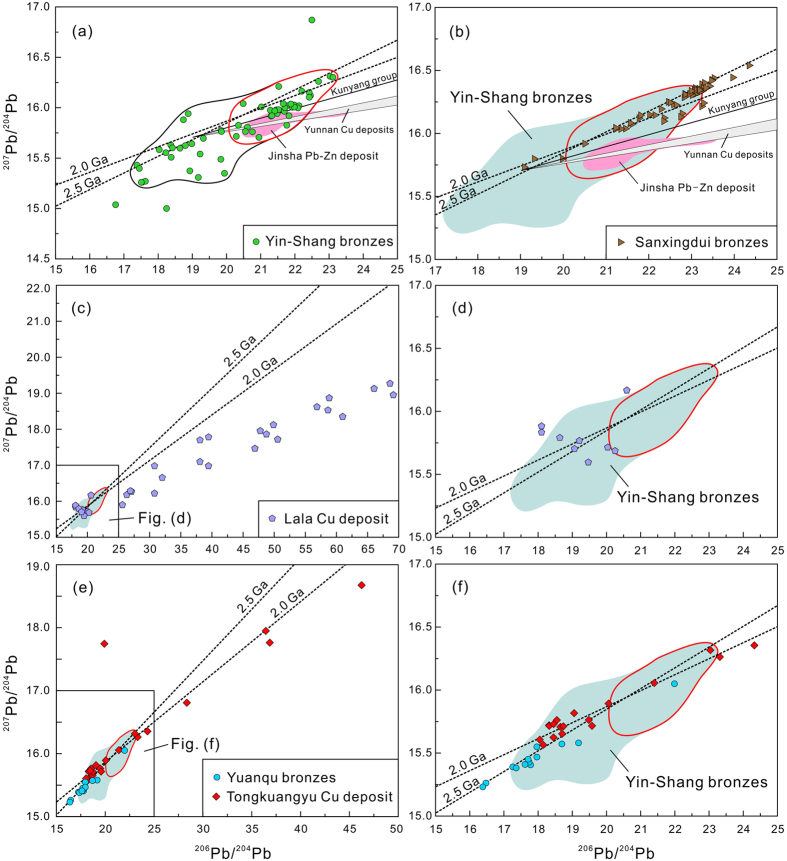
^260^Pb/^204^Pb versus ^207^Pb/^204^Pb diagram for ancient bronzes^2^,^7^,^9^,^58^,^59^ and ore deposits^60^ from China. Yin-Shang bronzes (black line marks the field for all Yin-Shang bronze samples so far published, red line marks the field for bronze with highly radiogenic lead isotopes) and Jinsha Pb-Zn deposit (**a**), Sanxingdui bronzes (**b**), Lala copper deposit in southwestern China, at the east margin of the Tibetan Plateau (**c,d**) and copper ores from Tongkuangyu deposit^22^ and bronzes from Yuanqu in central China^24^,^26^ (**e,f**) (Supplementary Table). The 2.0 Ga isochron line is defined by South Africa bronze (Fig. 4), the 2.5 Ga line is defined by Sanxingdui bronzes.

The problem that puzzled archaeologists for years is why such well-developed bronze technology all appeared so suddenly in the alluvial plain of the Yellow River and also several locations in southern China? More importantly, the lead isotopes of the early Yin-Shang bronzes are highly radiogenic^1^,^2^,^6^,^7^,^8^ and distinctively different from any of the known lead-zinc or tin deposits in China^9^. They are also different from most bronzes found in other places worldwide^10^,^11^,^12^,^13^,^14^,^15^,^16^,^17^. It has been proposed that the Yin-Shang people might have gotten raw materials from southwestern China, e.g., Jinsha for lead and Lala for copper, because the Sanxingdui bronzes from there all have essentially the same highly radiogenic lead isotopes as those of early Yin-Shang (Fig. 1a,b)^2^. The lead isotopes of Jinsha and other lead-zinc deposits in southwestern China, however, have considerably younger lead-lead isochron ages than those of the Yin-Shang and Sanxingdui bronzes (Fig. 1a,b). Also, the Lala copper deposit at the eastern margin of the Tibetan Plateau has an even more radiogenic lead than Yin-Shang bronze and plots along a much younger isochron line (Fig. 1c,d). Yet, significantly, Sanxingdui bronzes in southern China also appeared suddenly at roughly the same time as or slightly earlier than Yin-Shang bronzes.

Lead isotopes can be very good geochemical fingerprints for tracing the source of metals, but some precautions are needed before using such data to identify the original ores. Lead isotopic compositions of bronzes may be changed by a variety of metallurgical processes, for example by the mixing of different alloys, the use of additives during smelting, metal recycling and possibly Pb isotopic fractionation during smelting^18^. The first three processes homogenize lead isotopes, which could erase original unique signatures, such that the resulting isotopic composition of the bronzes becomes irrelevant to interpreting highly radiogenic lead discussed in this contribution. Lead acquired from additives, such as fluxes and foreign materials, may actually dominate the lead isotopic composition of the bronze, depending on the relative lead contents. For the last process, experiments have now shown that lead isotope fractionation during smelting is very small^18^,^19^,^20^, following the Rayleigh fractionation law, and thus would have only a negligible effect on the Yin-Shang bronzes even after repeated processing^21^.

## Tongkuangyu copper deposit

To our knowledge, the Tongkuangyu copper deposit is the only ore deposit so far discovered in China that has highly radiogenic lead isotopes^22^ as shown in a ^206^Pb/^204^Pb versus ^207^Pb/^204^Pb diagram. However, compared to most Yin-Shang bronzes, the lead isotopic compositions of analyzed Tongkuangyu ore samples (pyrite and chalcopyrite) are much more scattered with very low lead contents (Fig. 1e,f). Furthermore, when plotted on a ^206^Pb/^204^Pb versus ^207^Pb/^204^Pb diagram, Tongkuangyu ore samples plot along a slightly younger isochron than Yin-Shang bronze (Fig. 1e,f). Nevertheless, Tongkuangyu has been proposed as the supplier of both the copper and lead in Yin-Shang bronzes^23^,^24^, although it plots along a slightly younger line (Fig. 1e,f).

**Figure 2:**
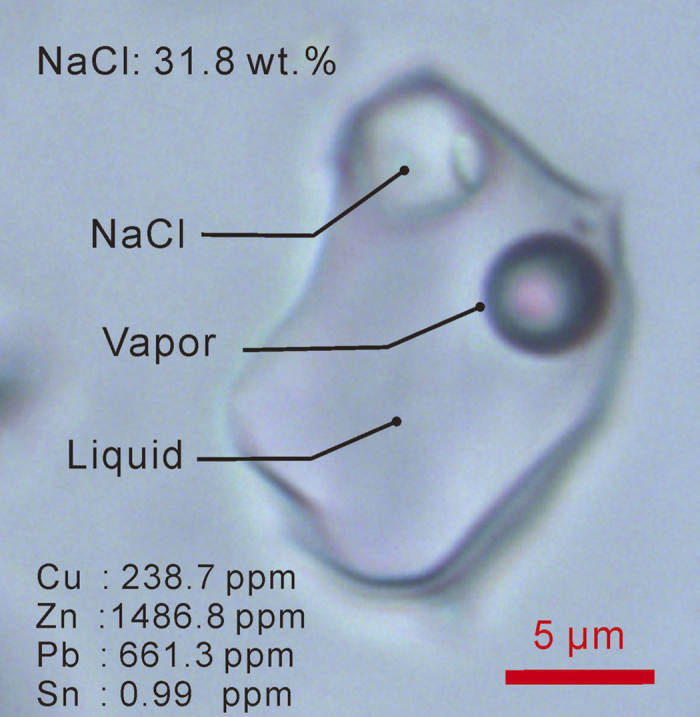
Copper, lead, zinc and tin concentrations and salinity of fluid inclusion from a quartz vein in the Tongkuangyu copper deposit. Analyzed using laser ablation inductively coupled mass spectrometer (LA-ICP-MS)^61^.

Tongkuangyu means “copper deposit valley” in Chinese. The mine is located in the Zhongtiao Mountains, Yuanqu County, Shanxi Province, central China^25^, which is about 250 kilometers to the west of Anyang, the ancient capital of Yin-Shang (Fig. 2). It belonged to the ancient state “Yuan”, affiliated with Yin-Shang^23^. A Shang dynasty copper smelting/foundry site was found and excavated in Yuanqu near Tongkuangyu^24^,^26^. As shown in Fig. 1e,f, most of the samples from this smelting site have distinctively less radiogenic lead than the Tongkuangyu ore or the Yin-Shang bronzes. Only one bronze found at the smelter plots marginally in the highly radiogenic lead field defined by early Yin-Shang bronze. In contrast, all smelting slags are less radiogenic. Therefore, this bronze with highly radiogenic Pb might have been taken to this site, rather than produced locally.

In the past experts on isotope geochemistry have excluded the copper ores at Tongkuangyu as a potential source of raw materials used to make Yin-Shang bronze because of the low lead contents in the copper ore^9^. Yin-Shang bronze has lead contents ranging from several hundred parts per million up to several weight percent^1^. In contrast, the lead content in Tongkuangyu copper ore is very low, i.e. present only in low parts per million levels. It has been estimated that for these copper ores, more than 80% of the lead must be of external sources to explain even the low lead content Yin-Shang bronzes. The addition of such large proportions of external lead would easily have erased the highly radiogenic lead isotopic characteristics of the Tongkuangyu ores^9^. Such a copper deposit alone obviously cannot make Yin-Shang bronzes.

Here we report that fluid inclusions in quartz from the Tongkuangyu deposit have high lead and zinc with low tin concentrations (Fig. 2). Given that galena and sphalerite usually precipitate at temperatures lower than those of chalcopyrite^27^,^28^, the lead contents in the Tongkuangyu deposit is likely to have increased with decreasing formation temperatures. Consequently, we can speculate that there might have been lead-(zinc) orebodies at shallower depths, which were already mined and removed by the ancient Shang people. This is seemingly supported by the bronzes with high lead contents in the Shang dynasty copper smelting/foundry site near Tongkuangyu^24^,^26^. The problem is that these Yuanqu bronzes from the smelting site have systematically less radiogenic lead isotopes^23^ (Fig. 1e,f). In addition, the lead and zinc concentrations in the Tongkuangyu fluid inclusions are both very high with very low tin, whereas Yin-Shang bronzes have very low zinc contents coupled with high lead and tin.

One way that separates lead from zinc is the smelting process. Copper - bronze experiments have demonstrate that Zn is recovered both in slags and in the vapor phase, but much less in the bronze metal phase^29^. Zinc is highly volatile with boiling temperature at 907 °C, which is lower than the melting temperatures of some bronze (~800–950 °C). Therefore, zinc is subjected to preferential volatile loss during smelting. Nonetheless, zinc in alloys behaves differently. For example, the melting point of Cu-Zn alloy goes up to 1000 °C, whereas Cu-Zn alloy first appeared around 1000 BC. For comparison, arsenic (with sublimation temperature at 615 °C) is more volatile than Zn, yet, most of the early bronzes contain percent levels of arsenic. Therefore, the key factor that controls zinc contents in bronze is the smelting process. Zinc may have suffered severe volatile loss before it alloyed with lead and copper.

## Tin and bronze technique

Tin is a more serious problem. Yin-Shang bronzes are mostly tin-copper alloys^1^. From where did the tin come? Tongkuangyu ore fluids are highly oxidized^25^, whereas tin is usually associated with reducing geological environments^30^, such that there should not be much tin in the Tongkuangyu ore deposit. This is supported by the low tin concentrations in the Tongkuangyu fluid inclusions (Fig. 2). Therefore, even if lead indeed came from Tongkuangyu, we still need to find out the source of tin. There is actually no known tin deposits within 1000 kilometers of Yin-Shang.

**Figure 3:**
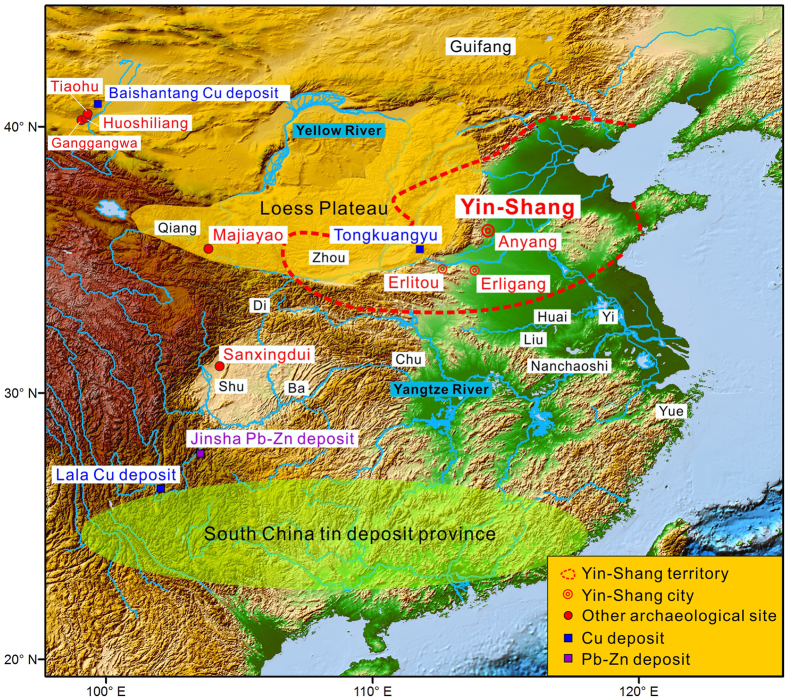
Map of the Yin-Shang Kingdom. Also shown are the distribution of tin deposits^30^, the Loess Plateau and places where ancient bronzes have been found in China^2^,^24^,^34^. Map used part of the map “color_etopo1_ice_full.tif.zip” from NOAA maps at: http://www.ngdc.noaa.gov/mgg/global/relief/ETOPO1/image/. Image created by J. Varner and E. Lim, CIRES, University of Colorado at Boulder.

China currently possess over 20 percent of the world’s total tin reserves, which are mostly located in far southern China (Fig. 3) to the south of the Yangtze River. This region was occupied by Baiyue and other tribes/states during the Ying-Shang period. These tin deposits were formed in the Mesozoic, such that the lead isotopic compositions of these tin deposits plot along a much younger isochron in a ^206^Pb/^204^Pb versus ^207^Pb/^204^Pb diagram. Moreover, cassiterite, the main tin ore mineral, almost universally has a low lead content. Therefore, tin deposits in South China cannot explain the highly radiogenic lead isotopic characteristics of Yin-Shang bronzes.

To make their bronzes, Yin-Shang people would have had to get tin from far southern China or elsewhere (Fig. 3). The Shang dynasty territory was very small at its beginning, and while expanding dramatically during the Yin-Shang period, it did not extend south of the Yangtze River even in its heyday.

It is true that long distance trade started very early in the history of civilizations. In particular, Yin-Shang people were famous for commerce. Indeed, “Shang” means “trade” or “commerce” in Chinese. So, it is possible that the Yin-Shang people get access to the needed tin through trade with tribes in southern China. The mystery is that if the Yin-Shang people had no local access to tin resources, how did they invent tin-bronze suddenly and independently in the first place and make the best bronzes in the world’s history?

Bronze was first produced in Mesopotamia at ~5000 BC or earlier^31^, which is more than 3500 years earlier than Yin-Shang bronzes. It started with arsenic bronze and then changed to tin bronze. In Mesopotamia, arsenic alloys with an arsenic concentration up to 5% were generally in use by the end of the 4^th^ and at the beginning of the 3^rd^ millennium BC, while tin bronzes were introduced during the middle of the 3^rd^ millennium^32^. In Europe, fahlore with high arsenic contents has been at least heated if not smelted in the second half of the 5^th^ millennium B.C. in the Tyrolean Alps, followed by popular usage of fahlore in the early Bronze Age^13^. In contrast, the Yin-Shang civilization started directly with tin bronzes. No arsenic bronze has ever been reported in China. This also strongly suggests that Yin-Shang bronze technology was likely imported into China.

All these suggest that the Yin-Shang people may have learned bronze technology elsewhere and brought it to China. Such a hypothesis, however, is not yet generally accepted, although an increasing amount of archaeological findings in northwestern China supports early culture and technology connections between China and the West^26^,^33^. The majority of archaeologists in China strongly insist, however, that although bronze was produced in China much later than in Mesopotamia, Egypt and several other regions, the bronze technology was developed independently in China.

## Ancient bronze in China

There are an increasing numbers of bronze objects older than the Shang Dynasty being unearthed in China, mostly in northwestern China. For example, a ca. 3000 BC bronze knife was discovered in Majiayao, Gansu Province, western China, about 1000 kilometers to the west of Anyang (Fig. 3). It is controversial whether the Majiayao knife marks the beginning of bronze production in China. First of all, it is an isolated bronze item, and no associated smelting site has been reported. Moreover, Majiayao is located in the west part of the Loess Plateau, which is covered by thick Quaternary loess with limited access to hard rocks. No necessary ore deposit near the Majiayao site has been reported, either. Therefore, we argue that the Majiayao bronze knife was most likely “imported”. Slightly younger bronzes perhaps as early as 2135 BC have been reported in Ganggangwa and Huoshiliang, Gansu Province^34^. These sites are located to the west of the Loess Plateau, which is about 2000 km to the west of Anyang (Fig. 3). Most of the 2135 BC bronze pieces, with the exception of one sample have low radiogenic isotopic compositions similar to those of the modern Baishantang mine site at Dingxin, indicating a possible source of copper ore^34^ for these pieces. These lead isotopic compositions are dramatically different from the early Yin-Shang bronzes. Sample Ganggangwa 3S does have highly radiogenic lead and strontium isotopes, however, it plots in a much younger isochron in a ^206^Pb/^204^Pb versus ^207^Pb/^204^Pb diagram, which is distinctively different from those of Yin-Shang bronzes^34^. Furthermore, none of these above mentioned sites belonged to Yin-Shang, and, importantly, these places also did not have local access to tin deposits (Fig. 3).

Sanxingdui is another famous ancient civilization in China with marvelous bronzes. It is located in Guanghan County, north of Chengdu, Sichuan Province, in the Upper Yangtze River region^26^. Sanxingdui bronzes are generally taken as coeval with or slightly earlier than Yin-Shang^2^. In contrast to other archaeological sites, Sanxingdui was much closer to tin deposits in southwestern China. Remarkably, Sanxingdui bronzes have lead isotopes similar to those of Yin-Shang, which has been taken as key evidence indicating connections between Yin-Shang and Sanxingdui. It has been proposed that the Yin-Shang people might have had a trading route across the Qinling Mountains and gotten their tin from the Sanxingdui people^2^, through trade, war, or marriage. This postulation is supported seemingly by bronzes with similarly highly radiogenic lead isotopes found between Yin-Shang and Sanxingdui^2^,^35^. Alternatively, according to myths, the Sanxingdui people came originally from the sea. If this was the case, then they should have been able to sail along the Yangtze River, and thus might trade tin brought to Yin-Shang along the Yellow River. Evidence so far available, however, indicates that the Sanxingdui civilization was very isolated within the Sichuan basin. Although Chengdu was mentioned in “Shih Chi”, there is no record of Sanxingdui culture in oracles or ancient books. Nonetheless, a key problem here is that the source of the Sanxingdui lead also remains unknown.

It was proposed that the Sanxingdui people had local access to ore deposits with highly radiogenic lead. For examples, the Jinsha Pb-Zn deposit and the Lala copper deposit are located at the eastern margin of the Tibetan Plateau, several hundred kilometers to the south of Sanxingdui (Fig. 3). Both Jinsha and Lala have highly radiogenic lead isotopes. They, however, plot along younger isochrons in a ^206^Pb/^204^Pb versus ^207^Pb/^204^Pb diagram (Fig. 1a–d), such that they cannot be the source of the required lead ore materials.

## Similarity of lead isotopes between African and Yin-Shang bronzes

**Figure 4:**
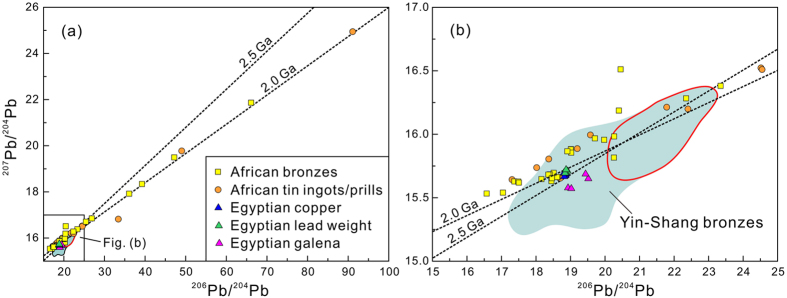
^206^Pb/^204^Pb versus ^207^Pb/^204^Pb diagram for ancient bronzes from China^2^,^7^,^9^ in comparison with tin ingots/prills, bronzes and slags from South Africa and Zimbabwe^36^, and from copper, lead and galena from the 18^th^ Dynasty of Ancient Egypt^42^. Yin-Shang bronzes, and Africa metals plot roughly along the same line. Metals from the 18^th^ Dynasty have homogenous lead isotopes, probably due to repeated recycling. Nonetheless, metals from Ancient Egypt plot in the field defined by Yin-Shang bronzes.

**Figure 5:**
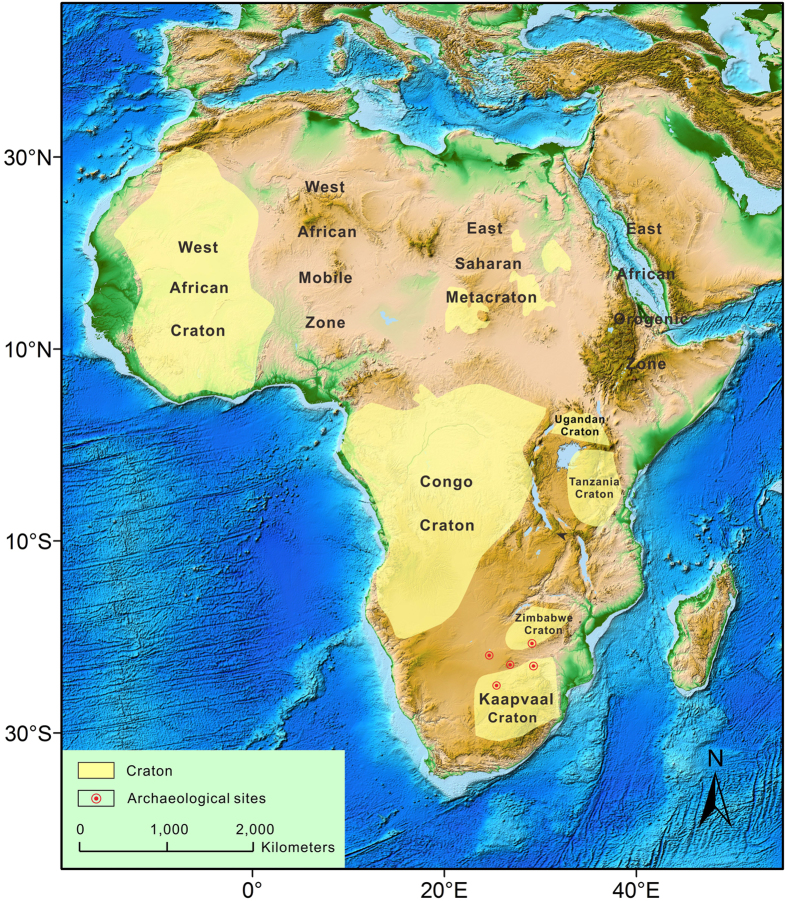
Sketched geologic map of Africa showing the distributions of Archean cratons, all of which have provide highly radiogenic “old” lead (2.0–2.5 Ga). Also shown are the locations of prehistory sites of tin ingots/prills, slag and broznes^36^ with highly radiogenic lead isotopes. In addition to Kaapvaal and Zimbabwe cratons, Congo, Tanzania and Uganda cratons are close the Nile River and thus were more accessible to ancient people. In addition, there are micro Archean cratons in the Sahara desert, which was more accessible in ancient time. This map used part of the map “color_etopo1_ice_full.tif.zip” from NOAA maps at: http://www.ngdc.noaa.gov/mgg/global/relief/ETOPO1/image/. Image created by J. Varner and E. Lim, CIRES, University of Colorado at Boulder.

Recent study show that “prehistory” tin ingots/prills and slages as well as bronzes from South Africa and Zimbabwe also have highly radiogenic lead isotopes, which do plot on an isochron very similar to the Yin-Shang bronzes (Fig. 4)^36^. These tin and bronze samples from South Africa and Zimbabwe are located between the Archean Kaapvaal and Zimbabwe cratons (Fig. 5).

In a ^206^Pb/^204^Pb versus ^207^Pb/^204^Pb diagram, bronzes from Yin-Shang, Sanxingdui, South Africa and Zimbabwe are all seen to plot along similar, very old isochrons of ~2.0–2.5 Ga (Figs 1 and 4), implying that these lead came from occurrences within ancient cratons. Both Africa and North China have Archean cratons older than 2.5 billion years and thus are in principle potential candidates for supplying the highly radiogenic lead of the Yin-Shang bronzes.

The North China Craton suffered multiple plate subductions^37^,^38^,^39^ and was eventually destroyed^40^ in the Early Cretaceous, likely due to ridge subduction^41^,^42^. The destruction of the North China Craton resulted in major magmatism^43^ and mineralization^41^,^44^, which obliterated most of the original Archean lead isotope signatures of the old craton. However, the Tongkuangyu copper deposit was already formed 1.9 billion years ago, and thus roughly preserved its older lead isotope characteristics. It is the only ore deposit so far reported in China with a lead isotopic composition and ischron age close to but slightly younger than those of early Yin-Shang bronzes. As discussed above, the Tongkuangyu copper deposit cannot be considered the single ore source for the early Yin-Shang bronzes because of its very low lead and tin contents. Neither does the Tongkuangyu deposit appear to have been the source of the systematically less radiogenic lead found in the bronzes at the Yuanqu smelting site near Tongkuangyu.

Tectonically, Tongkuangyu is located in the Paleo-Proterozoic Trans-North China Orogenic Belt formed through collision between the Eastern and Western Blocks of the North China Craton in the Paleo-Proterozoic era^45^. Convergent margins usually have very high oxygen fugacities, favorable for copper deposits^27^,^46^,^47^. This is supported by the high oxygen fugacity of Tongkuangyu. Tin deposits, on the other hand, are usually associated with reduced environments^30^ and thus the whole Trans-North China Orogenic Belt is unlikely to be favorable for tin deposits. In general, tin deposits are usually located several hundred kilometers away from convergent margins, e.g., as found in the South America and South China^30^,^48^,^49^. Furthermore, the North China Craton is a small block that is surrounded by subduction zones. In addition to the Paleo-Proterozoic Trans-North China Orogenic Belt, the Qinling-Dabie Orogenic Belt lies to the south^50^,^51^,^52^, the Central Asian Orogenic Belt lies to the north^53^ and the Pacific subduction zone lies to the east^39^,^42^,^54^. All these subductions face the North China Craton, and probably have elevated the oxygen fugacities, making them unfavorable sites for tin mineralization.

When putting together all the observations, the most straightforward conclusion is that both the Yin-Shang and the Sanxingdui bronzes were obtained in Africa, bearing the highly radiogenic lead isotopic signatures of the Africa Archean cratons. Alternatively, some ancient people might have come to China from Africa, carrying tin and/or bronzes with them.

South Africa and Zimbabwe are known for abundant archaeological sites^55^,^56^. Most archaeologists, however, consider these places to have been too far away from China for people to have been in contact in the Bronze Age. Even their relationship with ancient Egypt before ~1400 BC is not clear. Interestingly, so far published lead isotopic compositions of ancient Egyptian bronzes from the late Bronze Age are mostly less radiogenic^57^ than the Yin-Shang and the South African bronzes (Fig. 4). Mention was once made of some Ancient Egyptian bronzes have highly radiogenic lead isotopes, but that report did not show supporting data^9^. Nevertheless, the mystery remains as to how the Yin-Shang people would have gotten bronzes from these places?

In any case the lead isotopic signature of the Yin-Shang bronzes suggests that the ore deposits supplying their lead were most likely located in Archean cratons. The Africa continent is made up of several large Archean cratons (Fig. 5).The Congo, Tanzania and Uganda Cratons are essentially of the same age as the Kaapvaal and Zimbabwe Cratons. They would also be expected to have lead ore deposits with Archean age lead signatures. These cratons lie much closer to the Nile, and parts of them once even belonged to ancient Egypt during its early history. Might not the Yin-Shang people have gotten bronzes and/or raw materials from these places through trade or by other means at this time? During the late Dynasties, ancient Egypt lost access to these cratonic deposits, because of its shrinking territory, such that Egyptian bronzes in the 18^th^ Dynasty or later have less radiogenic lead isotopes (Fig. 4). The ancient Egyptian people may have also gotten lead ore from Saharan deposits. In addition to the large Archean cratons, there are several small Archean cratons within the Saharan metacraton (Fig. 5). Ore deposits–later abandoned and are now buried in the desert and having lead with Archean isochron ages–could possibly have been associated with these Saharan micro-cratons.

Given that bronze is often recycled^18^, early bronzes in Egypt may have been re-smelted and later mixed with more normal lead, thus explaining the very homogenous, but still radiogenic lead isotopes of 18^th^ Dynasty coppers (Fig. 4). In contrast, the original, highly radiogenic isotopic signature of Early Yin-Shang bronzes is well preserved in Yin-Shang tombs. More archaeological evidence is needed to confirm any of the above speculations. Until then, whether and how the ancient people in China obtained bronzes with highly radiogenic lead isotopes from Africa remains a puzzle.

## Additional Information

**How to cite this article**: Sun, W.- *et al*. Origin of the mysterious Yin-Shang bronzes in China indicated by lead isotopes. *Sci. Rep.* **6**, 23304; doi: 10.1038/srep23304 (2016).

## Supplementary Material

Supplementary Information
