# Supplementary material for: Origin of the mysterious Yin-Shang bronzes in China indicated by lead isotopes
Source: Sci Rep. 2016 Mar 18;6:23304. doi: 10.1038/srep23304 (PMC4796894; doi:10.1038/srep23304)
Supplement: Supplementary Information [file srep23304-s1.docx]

**Origin of the mysterious Yin-Shang bronzes in China indicated by lead isotopes**

Wei-dong Sun^1,2†^, Li-peng Zhang^1^, Jia Guo^1^, Cong-ying Li^1^, Yu-hang Jiang^1^, Robert, E. Zartman^1^ and Zhao-feng Zhang^3^

1. CAS Key Laboratory of Mineralogy and Metallogeny, Guangzhou Institute of Geochemistry, Chinese Academy of Sciences, Guangzhou 510640, China
2. CAS Center for Excellence in Tibetan Plateau Earth Sciences, Chinese Academy of Sciences, Beijing, 100101 China.
3. State Key Laboratory of Isotope Geochemistry, Guangzhou Institute of Geochemistry, Chinese Academy of Sciences, Guangzhou 510640, China

| Sample no. | | Sample type | | | ^207^Pb/^206^Pb | | | ^208^Pb/^206^Pb | | | ^206^Pb/^204^Pb | | | ^207^Pb/^204^Pb | | | ^208^Pb/^204^Pb | | | References | | |  |
| --- | --- | --- | --- | --- | --- | --- | --- | --- | --- | --- | --- | --- | --- | --- | --- | --- | --- | --- | --- | --- | --- | --- | --- |
| Yinshang bronzes | |  | | |  | | |  | | |  | | |  | | |  | | |  | | |  |
| 1 | | gu | | | 0.8081 | | | 2.0754 | | | 19.230 | | | 15.540 | | | 39.910 | | |  | | |  |
| 2 | | gu | | | 0.7592 | | | 2.0053 | | | 20.760 | | | 15.760 | | | 41.630 | | |  | | |  |
| 3 | | gu | | | 0.7828 | | | 2.0288 | | | 20.490 | | | 16.040 | | | 41.570 | | |  | | |  |
| 4 | | qufangyi | | | 0.7529 | | | 1.9545 | | | 21.530 | | | 16.210 | | | 42.080 | | |  | | |  |
| 5 | | double ears bronzes | | | 0.7498 | | | 1.9333 | | | 22.500 | | | 16.870 | | | 43.500 | | |  | | |  |
| 6 | | bronze pieces | | | 0.8508 | | | 2.1301 | | | 18.370 | | | 15.630 | | | 39.130 | | | Jin Zhengyao, 1987 | | |  |
| 7 | | bronze pieces | | | 0.8705 | | | 2.1306 | | | 17.530 | | | 15.260 | | | 37.350 | | |  | | |  |
| 8 | | bronze pieces | | | 0.8661 | | | 2.1339 | | | 17.630 | | | 15.270 | | | 37.620 | | |  | | |  |
| 9 | | bronze pieces | | | 0.8474 | | | 2.1062 | | | 18.740 | | | 15.880 | | | 39.470 | | |  | | |  |
| 10 | | bronze jue | | | 0.8443 | | | 2.0953 | | | 18.880 | | | 15.940 | | | 39.560 | | |  | | |  |
| 11 | | bronze pieces | | | 0.8715 | | | 2.1502 | | | 17.510 | | | 15.260 | | | 37.650 | | |  | | |  |
| 12 | | tin bronze | | | 0.7471 | | | 1.9396 | | | 21.305 | | | 15.916 | | | 41.323 | | |  | | |  |
| 13 | | lead-tin bronze | | | 0.7290 | | | 1.9354 | | | 21.984 | | | 16.027 | | | 42.547 | | |  | | |  |
| 14 | | tin bronze | | | 0.7359 | | | 1.9228 | | | 21.692 | | | 15.963 | | | 41.710 | | |  | | |  |
| 15 | | tin bronze | | | 0.7420 | | | 1.9321 | | | 21.484 | | | 15.942 | | | 41.509 | | | Tian et al., 2012 | | |  |
| 16 | | tin bronze | | | 0.8122 | | | 2.0301 | | | 19.325 | | | 15.695 | | | 39.232 | | |  | | |  |
| 17 | | tin bronze | | | 0.7082 | | | 1.9134 | | | 23.031 | | | 16.311 | | | 44.067 | | |  | | |  |
| 18 | | lead-tin bronze | | | 0.7241 | | | 1.9039 | | | 22.122 | | | 16.019 | | | 42.117 | | |  | | |  |
| YX001 | | knife | | | 0.7165 | | | 1.9156 | | | 22.693 | | | 16.260 | | | 43.472 | | |  | | |  |
| YX002 | | falchion | | | 0.7615 | | | 1.9809 | | | 21.023 | | | 16.008 | | | 41.643 | | |  | | |  |
| YX003 | | axe | | | 0.7360 | | | 1.9392 | | | 21.766 | | | 16.020 | | | 42.209 | | | Liu Jianyu, 2015 | | |  |
| YX004 | | axe | | | 0.7053 | | | 1.8670 | | | 23.114 | | | 16.302 | | | 43.153 | | |  | | |  |
| YX005 | | axe | | | 0.7178 | | | 1.9173 | | | 22.457 | | | 16.120 | | | 43.057 | | |  | | |  |
| YX006 | | axe | | | 0.7496 | | | 1.9523 | | | 20.951 | | | 15.706 | | | 40.901 | | |  | | |  |
| Sample no. | | Sample type | | | ^207^Pb/^206^Pb | | | ^208^Pb/^206^Pb | | | ^206^Pb/^204^Pb | | | ^207^Pb/^204^Pb | | | ^208^Pb/^204^Pb | | | References | | |  |
| YX007 | | axe | | | 0.7950 | | | 2.0358 | | | 19.842 | | | 15.774 | | | 40.394 | | |  | | |  |
| YX008 | | copper adze | | | 0.7447 | | | 1.9449 | | | 21.456 | | | 15.979 | | | 41.730 | | |  | | |  |
| YX009 | | copper adze | | | 0.7424 | | | 1.9380 | | | 21.525 | | | 15.980 | | | 41.715 | | |  | | |  |
| YX010 | | copper adze | | | 0.7300 | | | 1.9177 | | | 21.925 | | | 16.004 | | | 42.045 | | |  | | |  |
| YX011 | | axe | | | 0.7241 | | | 1.9068 | | | 22.092 | | | 15.998 | | | 42.125 | | |  | | |  |
| YX012 | | falchion | | | 0.7777 | | | 1.9921 | | | 20.350 | | | 15.825 | | | 40.540 | | |  | | |  |
| YX013 | | arch bronze | | | 0.7669 | | | 1.9808 | | | 20.611 | | | 15.806 | | | 40.825 | | |  | | |  |
| YX014 | | arch bronze | | | 0.8220 | | | 2.0687 | | | 18.248 | | | 15.000 | | | 37.751 | | |  | | |  |
| YX015 | | taper bronze | | | 0.7746 | | | 1.9800 | | | 20.289 | | | 15.716 | | | 40.172 | | |  | | |  |
| YX016 | | falchion | | | 0.7279 | | | 1.9105 | | | 21.989 | | | 16.006 | | | 42.009 | | |  | | |  |
| YX017 | | arch bronze | | | 0.7697 | | | 1.9844 | | | 19.942 | | | 15.349 | | | 39.572 | | |  | | |  |
| YX018 | | buck head knife | | | 0.7292 | | | 1.9114 | | | 21.831 | | | 15.919 | | | 41.729 | | | Liu Jianyu, 2015 | | |  |
| YX019 | | dagger-axe | | | 0.7669 | | | 1.9737 | | | 20.555 | | | 15.764 | | | 40.569 | | |  | | |  |
| YX020 | | dagger-axe | | | 0.7808 | | | 1.9968 | | | 19.836 | | | 15.488 | | | 39.609 | | |  | | |  |
| YX023 | | rammer | | | 0.7204 | | | 1.9055 | | | 22.420 | | | 16.150 | | | 42.720 | | |  | | |  |
| YX024 | | copper adze | | | 0.7506 | | | 1.9551 | | | 21.283 | | | 15.975 | | | 41.609 | | |  | | |  |
| YX025 | | bronze kui | | | 0.7982 | | | 2.0227 | | | 19.178 | | | 15.308 | | | 38.791 | | |  | | |  |
| YX026 | | falchion | | | 0.7329 | | | 1.9261 | | | 21.881 | | | 16.036 | | | 42.146 | | |  | | |  |
| YX027 | | arch bronze | | | 0.7370 | | | 1.9304 | | | 21.710 | | | 16.000 | | | 41.909 | | |  | | |  |
| YX028 | | bell | | | 0.8239 | | | 2.0535 | | | 18.986 | | | 15.642 | | | 38.989 | | |  | | |  |
| YX029 | | chisel | | | 0.7279 | | | 1.9239 | | | 22.204 | | | 16.163 | | | 42.719 | | |  | | |  |
| YX030 | | spear | | | 0.8470 | | | 2.0874 | | | 18.420 | | | 15.601 | | | 38.449 | | |  | | |  |
| YX031 | | spear | | | 0.8435 | | | 2.0856 | | | 18.385 | | | 15.508 | | | 38.343 | | |  | | |  |
| YX032 | | dagger-axe | | | 0.8121 | | | 2.0391 | | | 18.929 | | | 15.373 | | | 38.597 | | |  | | |  |
| YX033 | | dagger-axe | | | 0.8367 | | | 2.0741 | | | 18.642 | | | 15.598 | | | 38.665 | | |  | | |  |
| Sample no. | | Sample type | | | ^207^Pb/^206^Pb | | | ^208^Pb/^206^Pb | | | ^206^Pb/^204^Pb | | | ^207^Pb/^204^Pb | | | ^208^Pb/^204^Pb | | | References | | |  |
| YX034 | | battle-axe | | | 0.7458 | | | 1.9406 | | | 21.412 | | | 15.968 | | | 41.552 | | |  | | |  |
| YX035 | | ce | | | 0.7271 | | | 1.9238 | | | 21.765 | | | 15.826 | | | 41.871 | | |  | | |  |
| YX036 | | arch bronze | | | 0.7940 | | | 2.0136 | | | 19.853 | | | 15.763 | | | 39.975 | | |  | | |  |
| YX037 | | arch bronze | | | 0.7405 | | | 1.9344 | | | 21.662 | | | 16.040 | | | 41.902 | | |  | | |  |
| YX038 | | arch bronze | | | 0.8973 | | | 2.1750 | | | 16.758 | | | 15.037 | | | 36.449 | | |  | | |  |
| YX039 | | arch bronze | | | 0.7464 | | | 1.9416 | | | 21.329 | | | 15.921 | | | 41.411 | | | Liu Jianyu, 2015 | | |  |
| YX040 | | arch bronze | | | 0.7182 | | | 1.8891 | | | 22.423 | | | 16.104 | | | 42.360 | | |  | | |  |
| YX041 | | horse head knife | | | 0.8641 | | | 2.1278 | | | 18.034 | | | 15.583 | | | 38.373 | | |  | | |  |
| YX042 | | knife | | | 0.8876 | | | 2.1616 | | | 17.384 | | | 15.430 | | | 37.576 | | |  | | |  |
| YX043 | | arch bronze | | | 0.8823 | | | 2.1545 | | | 17.452 | | | 15.398 | | | 37.601 | | |  | | |  |
| YX044 | | arch bronze | | | 0.8311 | | | 2.0689 | | | 18.799 | | | 15.624 | | | 38.892 | | |  | | |  |
| YX045 | | arch bronze | | | 0.8533 | | | 2.1151 | | | 18.228 | | | 15.553 | | | 38.554 | | |  | | |  |
| Sanxingdui Brozes | | |  | | |  | | |  | | |  | | |  | | |  | | |  | | |
| ZY-317 | | bronze mask | | | 0.7264 | | | 1.9301 | | | 22.293 | | | 16.194 | | | 43.028 | | |  | | |  |
| ZY-318 | | bronze mask | | | 0.7064 | | | 1.8989 | | | 23.137 | | | 16.344 | | | 43.935 | | |  | | |  |
| ZY-319 | | bronze mask | | | 0.7020 | | | 1.8947 | | | 23.267 | | | 16.340 | | | 44.084 | | |  | | |  |
| ZY-320 | | bronze mask | | | 0.7041 | | | 1.9069 | | | 23.189 | | | 16.327 | | | 44.219 | | |  | | |  |
| ZY-321 | | bronze mask | | | 0.7403 | | | 1.9485 | | | 21.776 | | | 16.121 | | | 42.431 | | |  | | |  |
| ZY-322 | | bronze mask | | | 0.7062 | | | 1.8989 | | | 23.099 | | | 16.313 | | | 43.863 | | |  | | |  |
| ZY-323 | | bronze mask | | | 0.7276 | | | 1.9312 | | | 22.226 | | | 16.201 | | | 42.923 | | | Jin et al., 1995 | | |  |
| ZY-324 | | bronze mask | | | 0.7052 | | | 1.8824 | | | 23.242 | | | 16.390 | | | 43.751 | | |  | | |  |
| ZY-329 | | standing man picture | | | 0.7235 | | | 1.9291 | | | 22.460 | | | 16.250 | | | 43.328 | | |  | | |  |
| ZY-330 | | standing man picture | | | 0.7187 | | | 1.9178 | | | 22.584 | | | 16.231 | | | 43.312 | | |  | | |  |
| ZY-338 | | bronze zun | | | 0.7474 | | | 1.9719 | | | 21.458 | | | 16.038 | | | 42.313 | | |  | | |  |
| ZY-339 | | bronze zun | | | 0.7511 | | | 1.9778 | | | 21.349 | | | 16.035 | | | 42.224 | | |  | | |  |
| Sample no. | | Sample type | | | ^207^Pb/^206^Pb | | | ^208^Pb/^206^Pb | | | ^206^Pb/^204^Pb | | | ^207^Pb/^204^Pb | | | ^208^Pb/^204^Pb | | | References | | |  |
| ZY-340 | | bronze lei | | | 0.7084 | | | 1.8903 | | | 23.073 | | | 16.345 | | | 43.615 | | |  | | |  |
| ZY-341 | | bronze lei | | | 0.7907 | | | 2.0257 | | | 19.982 | | | 15.800 | | | 40.478 | | |  | | |  |
| ZY-342 | | bronze lei | | | 0.6990 | | | 1.8846 | | | 23.427 | | | 16.375 | | | 44.151 | | |  | | |  |
| ZY-343 | | bronze lei | | | 0.7037 | | | 1.8780 | | | 23.307 | | | 16.401 | | | 43.771 | | |  | | |  |
| ZY-344 | | bronze zun | | | 0.6793 | | | 1.8573 | | | 24.349 | | | 16.540 | | | 45.223 | | |  | | |  |
| ZY-345 | | bronze zun | | | 0.7290 | | | 1.9433 | | | 22.147 | | | 16.145 | | | 43.038 | | |  | | |  |
| ZY-346 | | bronze zun | | | 0.7085 | | | 1.8919 | | | 23.091 | | | 16.360 | | | 43.686 | | |  | | |  |
| ZY-347 | | Bronze fangyi | | | 0.6861 | | | 1.8520 | | | 23.968 | | | 16.444 | | | 44.389 | | |  | | |  |
| ZY-348 | | No.1 sacred tree | | | 0.7077 | | | 1.8992 | | | 23.086 | | | 16.338 | | | 43.845 | | |  | | |  |
| ZY-349 | | No.1 sacred tree | | | 0.7069 | | | 1.8951 | | | 23.121 | | | 16.344 | | | 43.817 | | |  | | |  |
| ZY-350 | | No.1 sacred tree | | | 0.7028 | | | 1.8833 | | | 23.279 | | | 16.360 | | | 43.841 | | |  | | |  |
| ZY-351 | | No.1 sacred tree | | | 0.7056 | | | 1.8901 | | | 23.177 | | | 16.354 | | | 43.807 | | | Jin et al., 1995 | | |  |
| ZY-352 | | No.2 sacred tree | | | 0.7011 | | | 1.8962 | | | 23.338 | | | 16.362 | | | 44.254 | | |  | | |  |
| ZY-353 | | No.2 sacred tree | | | 0.7056 | | | 1.8901 | | | 23.177 | | | 16.354 | | | 43.807 | | |  | | |  |
| ZY-360 | | dagger-axe | | | 0.7554 | | | 1.9659 | | | 21.227 | | | 16.035 | | | 41.730 | | |  | | |  |
| ZY-361 | | dagger-axe | | | 0.8175 | | | 2.0680 | | | 19.325 | | | 15.798 | | | 39.964 | | |  | | |  |
| ZY-362 | | bronze yuan | | | 0.7165 | | | 1.9044 | | | 22.645 | | | 16.225 | | | 43.125 | | |  | | |  |
| ZY-363 | | bronze yuan | | | 0.7083 | | | 1.9052 | | | 23.019 | | | 16.304 | | | 43.856 | | |  | | |  |
| ZY-364 | | triangle bronze | | | 0.7141 | | | 1.8955 | | | 22.846 | | | 16.314 | | | 43.305 | | |  | | |  |
| ZY-365 | | wheel bronze | | | 0.7218 | | | 1.9254 | | | 22.505 | | | 16.244 | | | 43.331 | | |  | | |  |
| ZY-325 | | bronze skin | | | 0.7558 | | | 1.9768 | | | 21.228 | | | 16.044 | | | 41.964 | | |  | | |  |
| ZY-326 | | bronze zun | | | 0.7438 | | | 1.9647 | | | 21.610 | | | 16.074 | | | 42.457 | | |  | | |  |
| ZY-327 | | dragon-tiger zun | | | 0.6977 | | | 1.8868 | | | 23.240 | | | 16.215 | | | 43.849 | | |  | | |  |
| ZY-328 | | dragon-tiger zun | | | 0.7210 | | | 1.9261 | | | 22.362 | | | 16.123 | | | 43.071 | | |  | | |  |
| ZY-331 | | dragon-tiger zun | | | 0.6975 | | | 1.8873 | | | 23.284 | | | 16.241 | | | 43.944 | | |  | | |  |
| Sample no. | | Sample type | | | ^207^Pb/^206^Pb | | | ^208^Pb/^206^Pb | | | ^206^Pb/^204^Pb | | | ^207^Pb/^204^Pb | | | ^208^Pb/^204^Pb | | | References | | |  |
| ZY-332 | | dragon-tiger zun | | | 0.6986 | | | 1.8770 | | | 23.532 | | | 16.439 | | | 44.170 | | |  | | |  |
| ZY-333 | | pan | | | 0.7092 | | | 1.8984 | | | 22.783 | | | 16.158 | | | 43.251 | | |  | | |  |
| ZY-334 | | pan | | | 0.7091 | | | 1.8966 | | | 22.750 | | | 16.132 | | | 43.148 | | |  | | |  |
| ZY-335 | | cap | | | 0.7201 | | | 1.9252 | | | 22.354 | | | 16.097 | | | 43.036 | | |  | | |  |
| ZY-336 | | bronze lei | | | 0.6995 | | | 1.8488 | | | 23.480 | | | 16.424 | | | 43.410 | | |  | | |  |
| ZY-337 | | bronze lei | | | 0.6995 | | | 1.8490 | | | 23.473 | | | 16.419 | | | 43.402 | | |  | | |  |
| ZY-354 | | bronze yuan | | | 0.7134 | | | 1.9011 | | | 22.829 | | | 16.286 | | | 43.400 | | |  | | |  |
| ZY-355 | | bronze yuan | | | 0.7402 | | | 1.9576 | | | 21.810 | | | 16.144 | | | 42.695 | | | Jin et al., 1995 | | |  |
| ZY-356 | | bronze yuan | | | 0.7137 | | | 1.9085 | | | 22.825 | | | 16.309 | | | 43.562 | | |  | | |  |
| ZY-369 | | bronze yuan | | | 0.7105 | | | 1.9033 | | | 22.961 | | | 16.314 | | | 43.702 | | |  | | |  |
| ZY-357 | | dagger-axe | | | 0.7105 | | | 1.8934 | | | 22.993 | | | 16.337 | | | 43.535 | | |  | | |  |
| ZY-358 | | dagger-axe | | | 0.8240 | | | 2.0937 | | | 19.092 | | | 16.732 | | | 39.973 | | |  | | |  |
| ZY-359 | | dagger-axe | | | 0.7333 | | | 1.9242 | | | 22.022 | | | 16.149 | | | 42.375 | | |  | | |  |
| ZY-366 | | bronze statue | | | 0.7048 | | | 1.8928 | | | 23.188 | | | 16.343 | | | 43.890 | | |  | | |  |
| ZY-367 | | bronze statue | | | 0.7456 | | | 1.9873 | | | 21.570 | | | 16.083 | | | 42.866 | | |  | | |  |
| ZY-368 | | bronze statue | | | 0.7762 | | | 2.0162 | | | 20.507 | | | 15.918 | | | 41.346 | | |  | | |  |
| Yuanqu Bronzes | |  | | |  | | |  | | |  | | |  | | |  | | |  | | |  |
| YQ-01 | | bronze ware | | | 0.8655 | | | 2.1313 | | | 17.965 | | | 15.549 | | | 38.289 | | |  | | |  |
| YQ-02 | | smelting slag | | | 0.8663 | | | 2.1066 | | | 17.783 | | | 15.406 | | | 37.461 | | |  | | |  |
| YQ-03 | | smelting slag | | | 0.8122 | | | 2.0072 | | | 19.183 | | | 15.580 | | | 38.503 | | |  | | |  |
| YQ-04 | | smelting slag | | | 0.8917 | | | 2.1718 | | | 17.259 | | | 15.390 | | | 37.484 | | | Cui et al., 2012 | | |  |
| YQ-05 | | bronze ware | | | 0.8860 | | | 2.1570 | | | 17.360 | | | 15.381 | | | 37.446 | | |  | | |  |
| YQ-06 | | smelting slag | | | 0.8698 | | | 2.1258 | | | 17.737 | | | 15.428 | | | 37.706 | | |  | | |  |
| YQ-08 | | bronze ware | | | 0.7299 | | | 1.9390 | | | 21.991 | | | 16.050 | | | 42.641 | | |  | | |  |
| YQ-11 | | smelting slag | | | 0.8724 | | | 2.1376 | | | 17.710 | | | 15.451 | | | 37.858 | | |  | | |  |
| Sample no. | | Sample type | | | ^207^Pb/^206^Pb | | | ^208^Pb/^206^Pb | | | ^206^Pb/^204^Pb | | | ^207^Pb/^204^Pb | | | ^208^Pb/^204^Pb | | | References | | |  |
| YQ-12 | | smelting slag | | | 0.8328 | | | 2.0764 | | | 18.698 | | | 15.572 | | | 38.825 | | |  | | |  |
| YQ-13 | | smelting slag | | | 0.8610 | | | 2.1208 | | | 17.966 | | | 15.468 | | | 38.102 | | |  | | |  |
| YQ-14 | | smelting slag | | | 0.8746 | | | 2.1308 | | | 17.618 | | | 15.409 | | | 37.540 | | | Cui et al., 2012 | | |  |
| YQ-16 | | bronze jia | | | 0.9264 | | | 2.2319 | | | 16.474 | | | 15.262 | | | 36.768 | | |  | | |  |
| YQ-17 | | bronze jia | | | 0.9296 | | | 2.2335 | | | 16.383 | | | 15.231 | | | 36.592 | | |  | | |  |
| Egyptian materials | |  | | |  | | |  | | |  | | |  | | |  | | |  | | |  |
| UC5111 | | Galena | | | 0.8230 | | | 2.0262 | | | 18.928 | | | 15.578 | | | 38.352 | | |  | | |  |
| UC5112 | | Galena | | | 0.8019 | | | 2.0058 | | | 19.516 | | | 15.650 | | | 39.145 | | |  | | |  |
| UC5115 | | Galena | | | 0.8186 | | | 2.0228 | | | 19.023 | | | 15.572 | | | 38.480 | | |  | | |  |
| UC5121 | | Galena | | | 0.8071 | | | 2.0143 | | | 19.436 | | | 15.687 | | | 39.150 | | |  | | |  |
| E2712c | | lead weight | | | 0.8305 | | | 2.0619 | | | 18.916 | | | 15.710 | | | 39.003 | | |  | | |  |
| E2712b | | lead weight | | | 0.8321 | | | 2.0632 | | | 18.865 | | | 15.698 | | | 38.922 | | | Shortland, A. J., 2006 | | |  |
| E2712a | | lead weight | | | 0.8329 | | | 2.0638 | | | 18.843 | | | 15.694 | | | 38.888 | | |  | | |  |
| E2712d | | lead weight | | | 0.8330 | | | 2.0667 | | | 18.873 | | | 15.721 | | | 39.005 | | |  | | |  |
| 1921.1132 | | copper | | | 0.8312 | | | 2.0593 | | | 18.862 | | | 15.678 | | | 38.843 | | |  | | |  |
| 1935.595 | | copper | | | 0.8317 | | | 2.0596 | | | 18.844 | | | 15.673 | | | 38.811 | | |  | | |  |
| 1921.115 | | copper | | | 0.8307 | | | 2.0614 | | | 18.887 | | | 15.689 | | | 38.934 | | |  | | |  |
| 1927.4104A | | copper | | | 0.8325 | | | 2.0623 | | | 18.818 | | | 15.666 | | | 38.808 | | |  | | |  |
| 1924.77 | | copper | | | 0.8345 | | | 2.0690 | | | 18.809 | | | 15.696 | | | 38.916 | | |  | | |  |
| Tongkuangyu copper deposit | | | | |  | | |  | | |  | | |  | | |  | | |  | | |  |
| 930-2-9-1 | pyrite | | | 0.7922 | | | 1.9567 | | | 20.062 | | | 15.893 | | | 39.256 | | |  | | |  |  |
| 930-2-9-2 | pyrite | | | 0.8468 | | | 2.0424 | | | 18.450 | | | 15.624 | | | 37.682 | | |  | | |  |  |
| 930-2-9-3 | pyrite | | | 0.8420 | | | 2.0868 | | | 18.655 | | | 15.707 | | | 38.930 | | | Xu et al., 2005 | | |  |  |
| 930-2-9-5 | pyrite | | | 0.4038 | | | 1.5056 | | | 46.243 | | | 18.675 | | | 69.623 | | |  | | |  |  |
| 930-2106-3- | pyrite | | | 0.4821 | | | 1.5757 | | | 36.846 | | | 17.762 | | | 58.060 | | |  | | |  |  |
| Sample no. | | Sample type | | | ^207^Pb/^206^Pb | | | ^208^Pb/^206^Pb | | | ^206^Pb/^204^Pb | | | ^207^Pb/^204^Pb | | | ^208^Pb/^204^Pb | | | References | | |  |
| 930-2106-3 | chalcopyrite | | | 0.8369 | | | 2.0473 | | | 18.703 | | | 15.653 | | | 38.290 | | |  | | |  |  |
| 930-3-2 | pyrite | | | 0.6724 | | | 1.8603 | | | 24.325 | | | 16.355 | | | 45.253 | | |  | | |  |  |
| 930-5145-2 | pyrite | | | 0.8090 | | | 1.9861 | | | 19.484 | | | 15.763 | | | 38.697 | | |  | | |  |  |
| 930-5145-3 | chalcopyrite | | | 0.8575 | | | 2.0987 | | | 18.151 | | | 15.565 | | | 38.093 | | |  | | |  |  |
| 810-5139-2 | pyrite | | | 0.4925 | | | 1.5869 | | | 36.442 | | | 17.949 | | | 57.829 | | |  | | |  |  |
| 870-5141-6 | chalcopyrite | | | 0.8647 | | | 2.3790 | | | 18.048 | | | 15.606 | | | 42.937 | | |  | | |  |  |
| 810-5153-9 | chalcopyrite | | | 0.8912 | | | 1.9576 | | | 19.912 | | | 17.745 | | | 38.979 | | | Xu et al., 2005 | | |  |  |
| 870-5147-11 | pyrite | | | 0.5928 | | | 1.5143 | | | 28.355 | | | 16.808 | | | 42.937 | | |  | | |  |  |
| 870-5147-1 | quartze | | | 0.8301 | | | 2.0006 | | | 19.054 | | | 15.817 | | | 38.119 | | |  | | |  |  |
| 810-5153-13 | chalcopyrite | | | 0.6975 | | | 1.8224 | | | 23.315 | | | 16.263 | | | 42.489 | | |  | | |  |  |
| 810-5153-16 | chalcopyrite | | | 0.8027 | | | 1.9649 | | | 19.578 | | | 15.716 | | | 38.469 | | |  | | |  |  |
| 870-5147-10 | chalcopyrite | | | 0.7082 | | | 1.7888 | | | 23.039 | | | 16.317 | | | 41.212 | | |  | | |  |  |
| TSX-1 | chalcopyrite | | | 0.8579 | | | 2.0783 | | | 18.324 | | | 15.720 | | | 38.082 | | |  | | |  |  |
| TSX-2 | chalcopyrite | | | 0.8576 | | | 2.1012 | | | 18.326 | | | 15.716 | | | 38.507 | | |  | | |  |  |
| TSX-9 | chalcopyrite | | | 0.8385 | | | 2.0811 | | | 18.739 | | | 15.712 | | | 38.998 | | |  | | |  |  |
| TSX-4 | chalcopyrite | | | 0.8497 | | | 2.0959 | | | 18.549 | | | 15.762 | | | 38.876 | | |  | | |  |  |
| TSX-5 | pyrite | | | 0.7502 | | | 2.0232 | | | 21.401 | | | 16.056 | | | 43.298 | | |  | | |  |  |
| TSX-6 | chalcopyrite | | | 0.8523 | | | 2.1038 | | | 18.455 | | | 15.730 | | | 38.825 | | |  | | |  |  |
| Lala copper deposit | | | |  | | |  | | |  | | |  | | |  | | |  | | |  |  |
| 1 | chalcopyrite | | | 0.6006 | | | 1.5873 | | | 27.090 | | | 16.270 | | | 43.000 | | |  | | |  |  |
| 2 | chalcopyrite | | | 0.2893 | | | 0.7795 | | | 66.120 | | | 19.130 | | | 51.540 | | |  | | |  |  |
| 3 | pyrite | | | 0.6140 | | | 1.6657 | | | 26.350 | | | 16.180 | | | 43.890 | | |  | | |  |  |
| 4 | pyrite | | | 0.6204 | | | 1.7202 | | | 25.630 | | | 15.900 | | | 44.090 | | | Sun, et al., 2006 | | |  |  |
| 5 | pyrite | | | 0.5266 | | | 1.4701 | | | 30.800 | | | 16.220 | | | 45.280 | | |  | | |  |  |
| 6 | pyrite | | | 0.8209 | | | 2.0563 | | | 19.207 | | | 15.767 | | | 39.496 | | |  | | |  |  |
| Sample no. | | Sample type | | | ^207^Pb/^206^Pb | | | ^208^Pb/^206^Pb | | | ^206^Pb/^204^Pb | | | ^207^Pb/^204^Pb | | | ^208^Pb/^204^Pb | | | References | | |  |
| 7 | pyrite | | | 0.7746 | | | 1.8665 | | | 20.251 | | | 15.687 | | | 37.798 | | |  | | |  |  |
| 8 | pyrite | | | 0.8746 | | | 2.1201 | | | 18.103 | | | 15.833 | | | 38.380 | | |  | | |  |  |
| 9 | chalcopyrite | | | 0.7843 | | | 1.8849 | | | 20.035 | | | 15.714 | | | 37.764 | | |  | | |  |  |
| 10 | chalcopyrite | | | 0.7850 | | | 1.8786 | | | 20.595 | | | 16.167 | | | 38.689 | | |  | | |  |  |
| 11 | chalcopyrite | | | 0.3756 | | | 1.1362 | | | 47.805 | | | 17.956 | | | 54.316 | | |  | | |  |  |
| 12 | chalcopyrite | | | 0.2806 | | | 0.7153 | | | 68.676 | | | 19.271 | | | 49.127 | | |  | | |  |  |
| 13 | chalcopyrite | | | 0.3720 | | | 1.1764 | | | 46.951 | | | 17.466 | | | 55.235 | | |  | | |  |  |
| 14 | pyrite | | | 0.8010 | | | 1.9704 | | | 19.470 | | | 15.596 | | | 38.363 | | | Huang, et al., 2012 | | |  |  |
| 15 | chalcopyrite | | | 0.4486 | | | 1.3759 | | | 38.110 | | | 17.098 | | | 52.435 | | |  | | |  |  |
| 16 | pyrite | | | 0.3660 | | | 1.1612 | | | 48.798 | | | 17.862 | | | 56.662 | | |  | | |  |  |
| 17 | pyrite | | | 0.5513 | | | 1.4701 | | | 30.802 | | | 16.982 | | | 45.281 | | |  | | |  |  |
| 18 | chalcopyrite | | | 0.4644 | | | 1.3758 | | | 38.112 | | | 17.698 | | | 52.435 | | |  | | |  |  |
| 19 | pyrite | | | 0.6204 | | | 1.7202 | | | 25.630 | | | 15.900 | | | 44.090 | | |  | | |  |  |
| 20 | pyrite | | | 0.5266 | | | 1.4701 | | | 30.800 | | | 16.220 | | | 45.280 | | |  | | |  |  |
| 21 | pyrite | | | 0.7746 | | | 1.8665 | | | 20.251 | | | 15.687 | | | 37.798 | | |  | | |  |  |
| 22 | pyrite | | | 0.6140 | | | 1.6657 | | | 26.350 | | | 16.180 | | | 43.890 | | |  | | |  |  |
| 23 | pyrite | | | 0.8209 | | | 2.0563 | | | 19.207 | | | 15.767 | | | 39.496 | | |  | | |  |  |
| 24 | chalcopyrite | | | 0.6006 | | | 1.5873 | | | 27.090 | | | 16.270 | | | 43.000 | | |  | | |  |  |
| 25 | chalcopyrite | | | 0.4300 | | | 1.1169 | | | 39.490 | | | 16.982 | | | 44.105 | | | Wang Dan, 2013 | | |  |  |
| 26 | pyrite | | | 0.3157 | | | 0.8088 | | | 58.703 | | | 18.530 | | | 47.479 | | |  | | |  |  |
| 27 | pyrite | | | 0.3271 | | | 0.9078 | | | 56.930 | | | 18.620 | | | 51.683 | | |  | | |  |  |
| 28 | pyrite | | | 0.3629 | | | 1.1043 | | | 49.935 | | | 18.123 | | | 55.141 | | |  | | |  |  |
| 29 | chalcopyrite | | | 0.2893 | | | 0.7794 | | | 66.130 | | | 19.130 | | | 51.540 | | |  | | |  |  |
| 30 | chalcopyrite | | | 0.2806 | | | 0.7153 | | | 68.676 | | | 19.271 | | | 49.127 | | |  | | |  |  |
| 31 | chalcopyrite | | | 0.3756 | | | 1.1362 | | | 47.805 | | | 17.956 | | | 54.316 | | |  | | |  |  |
| Sample no. | | Sample type | | | ^207^Pb/^206^Pb | | | ^208^Pb/^206^Pb | | | ^206^Pb/^204^Pb | | | ^207^Pb/^204^Pb | | | ^208^Pb/^204^Pb | | | References | | |  |
| 32 | pyrite | | | 0.3660 | | | 1.1612 | | | 48.798 | | | 17.862 | | | 56.662 | | |  | | |  |  |
| 33 | molybdenite | | | 0.3500 | | | 0.8615 | | | 50.622 | | | 17.719 | | | 43.613 | | |  | | |  |  |
| 34 | molybdenite | | | 0.2738 | | | 0.6456 | | | 69.222 | | | 18.953 | | | 44.689 | | |  | | |  |  |
| 35 | molybdenite | | | 0.3004 | | | 0.7362 | | | 61.072 | | | 18.348 | | | 44.963 | | |  | | |  |  |
| 36 | chalcopyrite | | | 0.3203 | | | 0.7496 | | | 58.903 | | | 18.868 | | | 44.156 | | |  | | |  |  |
| 37 | chalcopyrite | | | 0.4503 | | | 1.1168 | | | 39.492 | | | 17.782 | | | 44.105 | | |  | | |  |  |
| 38 | ore | | | 0.8473 | | | 2.0937 | | | 18.636 | | | 15.791 | | | 39.019 | | |  | | |  |  |
| 39 | ore | | | 0.8235 | | | 2.0295 | | | 19.068 | | | 15.703 | | | 38.699 | | | Chen et al., 1992 | | |  |  |
| 40 | ore | | | 0.6051 | | | 1.6412 | | | 26.916 | | | 16.286 | | | 44.174 | | |  | | |  |  |
| 41 | ore | | | 0.5199 | | | 1.2613 | | | 32.037 | | | 16.655 | | | 40.408 | | |  | | |  |  |

1 Chen, H. S. & Ran, C. Y. The isotope geochemical characteristics of copper deposits in Kangdian Axis. *Beijing: Geology Publishing House*, 100 (1992).

2 Cui, J. F., Tong, W. H. & Wu, X. H. A study of lead isotope ratio for bronzes and slags from Yuanqu City site. *Cultural Relics.* **7***, 80-84* (2012).

3 Huang, C. J., Li, Z. Q., Wang, J. Z. & Wang, D. Metallogenic Epoch Analysis of Lala Iron-Oxide-Cu-Au-U Deposit. *Journal of Henan Normal University (Natural Science Edition).* **40***, 80-83* (2012).

4 Jin, Z. Y. A Study of the Mineral Resources of Tin Contained in the Central Plains Bronzes of the Later Period of the Shang Dynasty. *Journal of Dialectics of Nature.* **9***, 47-55* (1987).

5 Jin, Z. Y. *et al.* A Study on Lead Isotope Ratios of the Sanxingdui Pit-burial Bronzes. *Cultural Relics.* **465***, 80-85* (1995).

6 Liu, J. Y. Scientific Study on the Shang and Zhou Periods Bronzes unearthed from Northern Shanxi: Cultural connections between loess highland and Anyang in the late Shang dynasty. *University of Science and Technology Beijing*, 238 (2015).

7 Shortland, A. J. Application of lead isotope analysis to a wide range of late bronze age Egyptian meterials. *Archaeometry.* **48**, 657–669 (2006).

8 Sun, Y., Shu, X. L. & Xiao, Y. F. Isotopic geochemistry of the Lala copper deposit, Sichuan Province, China and its metallogenetic significance. *Geochimica.* **35,** 553-559 (2006).

9 Tian, J. H., Jin, Z. Y., Li, R. L., Yan, L. F. & Cui, J. Y. An Elemental and Lead-isotopic Study on Bronze Helmets from Royal Tomb No.1004 in Yin Ruins. *Archaeometry.* **52**, 92-99 (2012).

10 Wang, D. Stable isotope geochemistry research of Lala Iron Oxide-Cu-Au-U (IOCG) Deposit. *Chengdu University of Technology, 71* (2013).

11 Xu, W. X., Wang, L. M., Li, H. & Guo, X. S. Isotope Geochemistry of Copper Deposits in the Zhongtiao Mountain. *Acta Geoscientica Sinica.* **26**, 130-133 (2005).
